# Childhood obesity classification systems and cardiometabolic risk factors: a comparison of the Italian, World Health Organization and International Obesity Task Force references

**DOI:** 10.1186/s13052-017-0338-z

**Published:** 2017-02-04

**Authors:** Giuliana Valerio, Antonio Balsamo, Marco Giorgio Baroni, Claudia Brufani, Claudia Forziato, Graziano Grugni, Maria Rosaria Licenziati, Claudio Maffeis, Emanuele Miraglia Del Giudice, Anita Morandi, Lucia Pacifico, Alessandro Sartorio, Melania Manco, on the behalf of the Childhood Obesity Group of the Italian Society of Pediatric Endocrinology and Diabetology, Alberto Comici, Nicola Corciulo, Procolo Di Bonito, Stefania Di Candia, Adriana Franzese, Elisabetta Modestini, Giuseppe Morino, Beatrice Moro, Rita Tanas

**Affiliations:** 1 Department of Movement Sciences and Wellbeing, Parthenope University, Naples, Italy; 2 Department of Medical and Surgical Sciences, Pediatric Unit, Azienda Ospedaliero-Universitaria S.Orsola-Malpighi, Bologna, Italy; 3 Department of Experimental Medicine, Sapienza University of Roma, Rome, Italy; 4 Endocrinology and Diabetes, Department of Medical Sciences, University of Cagliari, Cagliari, Italy; 5 Endocrinology and Diabetes Unit, Bambino Gesù Children’s Hospital, Rome, Italy; 6 Azienda Unità Sanitaria Locale di Viterbo, Viterbo, Italy; 7 Department of Pediatrics, Santa Maria delle Grazie Hospital, Pozzuoli, Napoli Italy; 8 Division of Auxology, Italian Auxological Institute, Verbania, Italy; 9 Department of Pediatrics, AORN Santobono-Pausilipon, Naples, Italy; 10 Pediatric Diabetes & Metabolic Disorders Unit, Department of Surgical Sciences, Dentistry, Gynecology and Pediatrics, University of Verona, Verona, Italy; 11 Department of Woman, Child and General and Specialized Surgery, University of Campania “Luigi Vanvitelli”, Napoli, Italy; 12 Pediatric Diabetes & Metabolic Disorders Unit, Department of Life & Reproduction Sciences, University Hospital of Verona, Verona, Italy; 13 Department of Pediatrics and Child Neuropsychiatry, Sapienza University of Rome, Rome, Italy; 14 Division of Auxology, Italian Auxological Institute, Milan, Italy; 15 Research Unit for Multifactorial Diseases, Scientific Directorate, Bambino Gesù Children’s Hospital, IRCCS, Rome, Italy

**Keywords:** Adolescents, Body mass index, Cardiometabolic risk factors, Children, Classification, Cut-offs, Obesity, Overweight

## Abstract

**Background:**

Body Mass Index Italian reference data are available for clinical and/or epidemiological use, but no study compared the ability of this system to classify overweight and obesity and detect subjects with clustered cardiometabolic risk factors with international standards. Therefore our aim was to assess 1) the agreement among the Italian Society for Pediatric Endocrinology and Diabetology (ISPED), the World Health Organisation (WHO) and the International Obesity Task Force (IOTF) Body Mass Index cut-offs in estimating overweight or obesity in children and adolescents; 2) the ability of each above-mentioned set of cut-points to detect subjects with cardiometabolic risk factors.

**Methods:**

Data of 6070 Italian subjects aged 5–17 years were collected. Prevalence of normal-weight, overweight and obesity was determined using three classification systems: ISPED, WHO and IOTF. High blood pressure, hypertriglyceridemia, low high density lipoprotein-cholesterol and impaired fasting glucose were considered as cardiometabolic risk factors.

**Results:**

ISPED and IOTF classified more subjects as normal-weight or overweight and less subjects as obese as compared to WHO (*p* <0.0001) in the whole sample and in groups divided by gender and age. The strength of agreement between the three methods compared to each other was excellent for overweight (including obesity) definition (*k* > 0.900), while it differed for obesity definition, ranging from the highest agreement between ISPED and IOTF (*k* 0.875) to the lowest between ISPED and WHO (*k* 0.664). WHO had the highest sensitivity, while ISPED and IOTF systems had the highest specificity, in identifying obese subjects with clustered cardiometabolic risk factors. Analogous results were found in subjects stratified by gender or age.

**Conclusions:**

ISPED and IOTF systems performed similarly in assessing overweight and obesity, and were more specific in identifying obese children/adolescents with clustered cardiometabolic risk factors; on the contrary, the WHO system was more sensitive. Given the seriousness of the obesity epidemic, we wonder whether the WHO system should be preferable to the national standards for clinical practice and/or obesity screening.

## Background

The use of body mass index (BMI) to define overweight (OW) or obesity (OB) in children and adolescents is well established for both clinical and public health applications [1]. At present, the most widely used international growth charts in Europe [2, 3] are those proposed by the World Health Organization (WHO) in 2007 [4] and by the International Obesity Task Force (IOTF) in 2000 [5], updated in 2012 [6]. The WHO system uses arbitrarily chosen cut-points of BMI percentiles and, with regard to subjects from 5 to 17 years, is based on data issued before the obesity epidemic from the National Center for Health Statistics charts (NHES II and III and NHANES I) (1971–1974). Differently, the IOTF system uses smooth sex-specific BMI curves, constructed to match the values of 25 kg/m^2^ (OW) and 30 kg/m^2^ (OB) at 18 years, thus providing age and gender BMI cut-offs for OW and OB, and is based on large data sets from six countries or regions covering different races/ethnicities. Practically, the IOTF approach is founded on the idea that the BMI-based definitions of OW and OB at 18 years of age, which are considered to be associated with health consequences in adults, can be tracked back to younger ages.

National BMI reference data are available in many countries and their adoption is recommended for clinical and national epidemiological use [7]. In order to supply pediatricians with national based growth charts, reference values have been recently developed in Italy on data collected among school-children between 1990 and 2004. The first Italian reference charts for children aged 6 to 20 years were published by Cacciari et al. on behalf of the Italian Society for Pediatric Endocrinology and Diabetology (ISPED) in 2002 [8]. Successively these references were extended to preschool age [9], obtaining charts that apply to the Italian population from 2 to 20 year of age. Obviously, these references can over- or under-estimate the prevalence of OW and/or OB with respect to an hypothetical ideal gold-standard for the assessment of body fat, which indeed is lacking. It is more realistically worth for users of national standards to compare them with the international BMI systems conventionally accepted as reference, in order to be aware of their potential for misclassification.

To our knowledge, no previous study has compared the Italian approach to WHO or IOTF reference systems. Therefore the aim of this study was 1) to assess the agreement between the Italian system and the two most frequently employed international systems, the WHO and the IOTF, in classifying paediatric OW or OB, and 2) to evaluate the potential differences among the Italian and the international systems as regards the ability to detect subjects with clustered cardiometabolic risk factors (CMRFs).

## Methods

This study derives from a retrospective cross-sectional survey endorsed by the Childhood Obesity Group of ISPED designed to investigate the prevalence of the major CMRFs in outpatient children followed in specialist centers for the care of OW and OB in Italy. Seventeen obesity services (seven in northern, five in central and five in southern Italy) located in hospital or university hospital settings participated to the study, providing medical records of 6070 children and adolescents aged 5–17 years (3009 males, 3061 females) from 2003 to 2013. They were geographically distributed across the northern (*n* = 1304, 21.5%), central (*n* = 2454, 40.4%), and southern (*n* = 2312, 38.1%) Italian regions. The selection of centers was based on the following criteria: 1) specialized centers for the care of pediatric OW/OB; 2) availability of anthropometric data and CMRFs analyzed with standard methods; 3) centralized procedure for biochemical analysis in each center. The inclusion criteria for subjects were: European ancestry, age (5–17 years), and having complete data set. The exclusion criteria were: secondary OB, chronic diseases, malformations and chronic use of drugs leading to metabolic disturbances (such as steroids). The majority of OW or OB children were referred by their family pediatricians. To extend the range of body size, data about normal-weight (NW) children and adolescents (*n* = 1146) were derived from the following databases: 1) 508 subjects randomly selected from the registry database (Verona) [10]; 2) 272 children participating in a study on the risk of complex diseases in the Italian population (Rome, Bambino Gesù Children’s Hospital) [11]; 3) 157 healthy students selected from four schools to participate in a pilot study aimed at preventing CVD in childhood (Rome, La Sapienza) [12]; 4) 259 healthy outpatients evaluated for pre-operative assessment before minor surgery interventions (Santobono Pausilipon *n* = 52, and Santa Maria delle Grazie Pozzuoli Hospitals, *n* = 157) [13].

The study was approved by the Ethics Committee of the Second University in Naples, Italy (reference number 834/2016) and conformed to the guidelines of the European Convention of Human Rights and Biomedicine for Research in Children. The directive 95/46/EC of the European Parliament and of the Council of 24 October 1995 on the protection of personal data was complied with for data storage and handling in order to ensure patient data protection and confidentiality.

### Anthropometric and clinical assessment

Body weight was determined to the nearest 0.1 kg on accurate and properly calibrated standard beam scales, in minimal underclothes and no shoes. Height was measured to the nearest 0.5 cm on standardized, wall-mounted height boards according to standardized procedures [14]. The BMI was calculated as weight divided by square of height (kg/m^2^). Height and weight were measured by one investigator in each centre, who was specifically trained in anthropometry; the average of the two closest measurements of height was used for the analysis; if a difference of 0.5 cm or more was found, a third measurement was taken and the median was calculated.

Blood pressure was measured according to a standardized protocol [15]. Briefly, the cuffs had bladders long enough to encircle at least one-half of the upper arm without overlapping and widths that covered at least two-thirds of the upper arm. The average of three blood pressure values was used for analysis.

### Biochemical parameters

Fasting venipuncture samples were drawn for triglycerides, high-density lipoprotein–cholesterol and glucose measurements and analyzed with standard techniques. Although analyses were performed in different laboratories, all centres belong to the Italian National Health system and undergo to semi-annual quality controls and inter-lab comparisons, contributing to limit the potential differences among laboratories.

### Case definitions

Each subject was classified as NW, OW or OB by comparing his/her BMI with ISPED or WHO percentiles for age and sex. According to the ISPED system, the BMI value ≥ 5th percentile and < 75th was considered as NW, the BMI ≥ 75th and < 95th percentile was considered as OW and the BMI ≥95th percentile was considered as OB [8]; according to the WHO system the BMI value ≥ 5th percentile and < 85th was considered as NW, the BMI ≥ 85th and < 97th percentile was considered as OW and the BMI ≥97th percentile was considered as OB [4]. As regards the Cole’s approach, each subject was classified as NW, OW or OB when his/her BMI cut points was equal to or greater than the value plotted on the sex related curves crossing a BMI of 18.5, 25 and 30 kg/m^2^ at the age of 18, respectively. Subjects were categorized in two age groups: children (5–9.9 years) and adolescents (10–17.9 years).

The following CMRFs were considered: high blood pressure (systolic and/or diastolic blood pressure ≥95th percentile for age, sex and height) [15]; high triglycerides (≥100 mg/dL between 0 and 9 years and ≥ 130 md/dL between 10 and 19 years) [16]; low high-density lipoprotein–cholesterol (<40 mg/dL) [16].

### Statistical analysis

Continuous data are reported as means and standard deviations, with categorical data as counts and percentages. Variables not normally distributed (weight, BMI) were logarithmically transformed; for clarity of interpretation, results are expressed as untransformed values. Intergroup comparisons were made by the Student’s *t*-test. The prevalence of NW, OW and OB using the different classification systems was determined in the whole group and in each gender and age subgroups. The rate of agreement between the different criteria was measured by kappa (*k*) statistics that measures the agreement in individual levels by calculating *k* = (P_o_-P_e_)/(1-P_e_) where P_o_ = the observed probability of agreement and P_e_ = the probability of expected agreement by chance. *K* statistics was rated as follows: < 0 = less than chance agreement; 0.01–0.20 = slight agreement; 0.21–0.40 = fair agreement; 0.41–0.60 = moderate agreement; 0.61–0.80 = substantial agreement; 0.81–0.99 = almost perfect agreement. To compare the criteria as for the NW, OW and OB prevalence, the paired McNemar test was used.

Logistic regression analysis was used to predict the likelihood of clustered CMRFs in BMI groups defined by classification systems, controlling for gender, age, and centers. Dummy variables were created to compute odds ratios (ORs) for these factors. The NW group was the reference group (OR = 1.00).

The diagnostic accuracy of the OB and of the OW cut-points to discriminate the presence of clustered CMRFs (≥2 risk factors) was assessed for the three systems in the whole population, and gender and age subgroups. As statistical approach, we assessed the sensitivity (proportion of subjects with clustered CMRFs who are OW (including OB) or OB), and specificity (proportion of subjects without clustered CMRFs who are NW or NW/OW).

The Statistical Package of Social Sciences (SPSS, Chicago, IL, USA) for Windows software program release 21.0 was used. A *p* value <0.05 was considered significant.

## Results

**Fig. 1:**
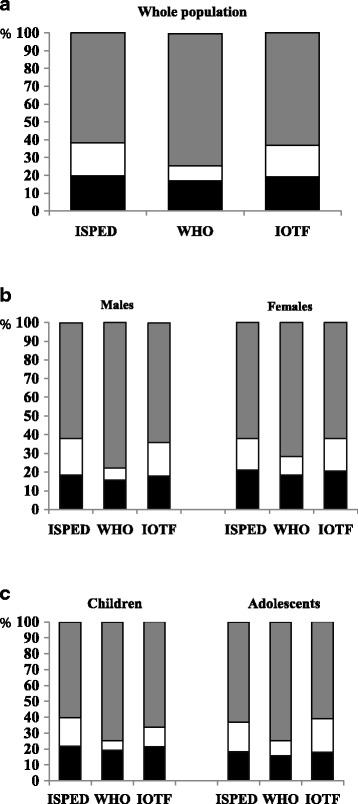
Distribution of subjects classified as normal weight (*black bars*), overweight (*white bars*) or obese (*grey bars*) according to ISPED, WHO and IOTF in the whole population (Panel **a**), and in groups divided by gender (Panel **b**), and age (Panel **c**)

The anthropometric characteristics of the study population are presented in Table 1, while the distribution of subjects classified as NW, OW or OB according to the different reference systems is shown in the Fig. 1.

**Table 1:** Anthropometric, clinical and biochemical characteristics of the whole study population and groups stratified by gender and age

		Gender		Age	
	Total	Males	Females	Children	Adolescents
Number	6070	3009	3061	2318	3752
Age (years)	11.8 ± 2.7	10.9 ± 2.6	10.7 ± 2.8	8.1 ± 1.2	12.45 ± 1.9
Height (cm)	145.9 ± 15.0	147.5 ± 15.4	144.3 ± 14.5	132.3 ± 9.7	154.3 ± 11.1
Weight (kg)	58.0 ± 23.0	59.7 ± 23.6	56.4 ± 22.4	41.9 ± 12.9	67.9 ± 22.3
Body Mass Index (kg/m^2^)	26.4 ± 6.7	26.6 ± 6.6	26.2 ± 6.8	23.6 ± 5.4	28.1 ± 6.9
Systolic blood pressure (mmHg)	110.3 ± 14.5	111.4 ± 14.7	109.1 ± 14.2	103.7 ± 12.5	114.3 ± 14.2
Diastolic blood pressure (mmHg)	66.2 ± 10.7	66.4 ± 10.7	66.1 ± 10.6	63.4 ± 10.0	68.0 ± 10.7
Glucose (mg/dL)	84.4 ± 8.6	85.2 ± 8.6	83.5 ± 8.5	75.2 ± 40.7	85.7 ± 46.9
Tryglicerides (mg/dL)	81.7 ± 44.9	80.9 ± 45.6	82.42 ± 44.3	52.1 ± 12.3	49.3 ± 12.6
HDL-Cholesterol (mg/dL)	50.4 ± 12.6	50.3 ± 12.9	50.5 ± 12.2	83.6 ± 8.1	84.8 ± 8.9

By considering either the whole population or groups stratified by gender and age, ISPED and IOTF classified more subjects as NW or OW and less subjects as OB as compared to WHO (p <0.0001). When the overweight threshold (including obesity) was used, the strength of agreement between the three methods compared each other was excellent; when the obesity threshold was used, the strength of agreement was quite excellent between ISPED and IOTF (*k* 0.873), substantial between ISPED and WHO (*k* 0.692), moderate between IOTF and WHO (*k* 0.731) (Table 2).

**Table 2:** Agreement (kappa coefficient and Standard Error) between the ISPED, WHO and IOTF references for the classification of participants according to the overweight or obesity thresholds

	Overweight	Obesity
ISPED vs WHO	0.906 (0.007)	0.692 (0.010)
ISPED vs IOTF	0.974 (0.004)	0.873 (0.006)
IOTF vs WHO	0.925 (0.006)	0.731 (0.009)

Standard error in brackets

All kappa coefficients were significant (*P* < 0.0001)

*ISPED* Italian Society for Pediatric Endocrinology and Diabetology, *WHO* World Health Organization, *IOTF* International Obesity Task Force

The OR for clustered CMRFs was separately calculated for each of the three reference systems. Compared with NW, OB subjects had higher risk of association with clustered CMRFs than OW subjects, independent of the classification system used (Table 3).

**Table 3:** Odds Ratios (95% CI) for the presence of at least one CMRF (among high Tg, low HDL-C and HBP) for each reference system, controlled for age, gender and center

	ISPED	WHO	IOTF
Normal weight	1	1	1
Overweight	3.481 (2.730–4.439)	2.686 (1.959–3.683)	3.934 (3.059–5.060)
Obesity	6.198 (4.898–7.843)	6.446 (5.009–8.295)	6.728 (5.276–8.579)

*ISPED* Italian Society for Pediatric Endocrinology and Diabetology, *WHO* World Health Organization, *IOTF* International Obesity Task Force

Sensitivity and specificity for predicting clustered CMRFs of categories of OW (including OB) or OB subjects defined by ISPED, WHO, or IOTF are synthetized in Table 4.

**Table 4:** Sensitivity and specificity of ISPED, WHO, and IOTF defined categories of overweight (including obesity) or obesity for predicting clustered CMRFs in the total sample and in groups stratified by gender and age

Overweight					
	Total	Gender		Age	
		Males	Females	Children	Adolescents
ISPED					
sensitivity	98.1	97.3	99.1	98.2	98.1
specificity	22.0	20.6	23.5	24.0	20.8
WHO					
sensitivity	98.9	98.4	99.4	98.6	98.9
specificity	19.1	17.7	20.5	21.1	17.8
IOTF					
sensitivity	98.4	97.9	99.1	98.2	98.5
specificity	21.6	20.1	22.9	23.6	20.3
Obesity					
	Total	Gender		Age	
		Males	Females	Children	Adolescents
ISPED					
sensitivity	86.3	85.0	87.8	87.7	85.7
specificity	41.3	41.4	41.1	42.6	40.4
WHO					
sensitivity	96.0	95.4	96.7	97.7	95.3
specificity	28.0	24.7	31.2	27.7	28.2
IOTF					
sensitivity	87.2	86.4	88.2	93.2	84.5
specificity	40.1	39.1	41.0	36.5	42.4

*ISPED* Italian Society for Pediatric Endocrinology and Diabetology, *WHO* Word Health Organization, *IOTF* International Obesity Task Force

As regards the definition of OW (including OB), the three systems performed quite similarly. As regards the definition of OB, WHO had the highest sensitivity in identifying OB subjects with clustered CMRFs, while ISPED and IOTF systems performed similarly with a sensitivity of 86–87%. Analogous results were found in subjects stratified by gender or age.

## Discussion

This study compared the ability of a national BMI reference system for estimating OW and OB in children and adolescents with the two most frequently employed international systems, the WHO and IOTF systems, and demonstrated that there was a high agreement between the three classification methods in the estimated proportions of overweight (including obesity) prevalence. With specific regard to the obesity classification, instead, the highest prevalence of children and adolescents classified as OB was achieved using the WHO system, while the ISPED thresholds of BMI were similar to the IOTF system. This occurred in the total sample and in groups stratified by gender and age. In addition, this study compared the ability of each set of cut-point to screen subjects with CMRFs, demonstrating that there was no difference between ISPED and IOTF, while the WHO thresholds had higher sensitivity and lower specificity in identifying OB subjects with clustered CMRFs, with respect to the other systems.

IOTF or WHO standards are the two international systems employed in Europe to classify OW and OB in children and adolescents [2]. IOTF system is considered to be more biologically meaningful compared to the references based on statistical distribution (i.e. percentiles) [17]. Therefore, several international scientific societies, including the Italian Society of Pediatrics, recommended the use of IOTF not only for international descriptive and comparative purposes but also for diagnostic purposes, even though it was not proposed for assessing excess weight at the individual level [18–21]. Before the first Italian BMI charts were made available in 2002 for subjects from 6 to 20 years [8] and in 2006 for subjects from 2 to 20 years [9], Italian pediatricians were inevitably accustomed to use the international standards. Moreover, doubts about the use of the national charts were expressed since they were constructed upon data collected between 1996 and 2004, when the increase in OW/OB was going on in the Italian pediatric population. This concern limited the widespread use of the national charts, despite the recommendation to use national BMI reference data for the assessment of childhood obesity [22].

To our knowledge, no study has specifically compared the performance of the Italian system with the WHO or IOTF systems. ISPED thresholds tended to estimate a lower prevalence of OB subjects in both genders and different age-groups with respect to WHO. This finding is in agreement with a previous paper [9] comparing ISPED with other BMI systems, as CDC 2000 [23] and UK 90 [24] charts, and confirms that the 95th centile of the Italian BMI charts is higher than that of the other references. The agreement on OW classification was excellent by comparing the three systems each other, while it differed regarding OB classification: it was moderate by comparing ISPED versus WHO, and excellent by comparing ISPED versus IOTF. The almost perfect agreement between ISPED and IOTF in identifying children from 5 to 17 years with OW and OB shows that the thresholds set equal to the 75th or 95th centiles of ISPED charts as proposed by Cacciari et al. [9] match quite well the IOTF thresholds for OW or OB in this age range. Differently from our findings, previous studies comparing the IOTF reference with the BMI 85th and 95th percentiles from several countries, underlined that IOTF tended to underestimated obesity prevalence, while it gave similar estimates for overweight [25–29].

The emergence of the childhood obesity epidemic poses the challenge of assessing the presence of CMRFs already in children [30–32], which may influence the intensity of treatment [33]. Since the variety of statistical definitions of OW and OB obtained by the choice of one system instead of another can have clear implications for health resource planning [34–36] we also assessed the ability of the ISPED system to detect the association with CMRFs, in comparison with the other international systems. Our data show that, independently of the classification system used, OW, and even more consistently OB subjects, had significantly increased risk for the presence of clustered CMRFs with respect to NW subjects.

The strength of our study resides in the very large sample size, which allowed also subgroups stratification, in the measured rather than self-reported anthropometric data, and completeness of all the variables recorded. Our study has also some limitations. Firstly, OW/OB subjects were recruited in pediatric obesity services, and may be not representative of the general population. Limitations may also depend on the multicenter recruitment of our subjects. However, anthropometric and clinical data were collected according to standardized procedures, and inter-laboratory quality controls were regularly performed, as prescribed by Italian-law, so that precision and accuracy of anthropometric, clinical and biochemical analysis is guaranteed. In addition, the association between clustered cardiometabolic risk factors and classification of OW or OB was controlled by age, gender and center in the logistic regression analysis to mitigate effect, if there was any, of lack of centralized dosages. Lastly, the cross sectional design of the study does not allow assessing the ability of the BMI cut-offs to predict cardio-metabolic outcomes in adulthood.

## Conclusions

Our results highlight the differences in the agreement in OW and OB classification as well as and in the diagnostic accuracy of the associated CMRFs that may arise using national or international BMI reference data. These differences are explained by population variations in the pattern of BMI with age and gender between nations and/or the time of data collection. The use of the IOTF system matches quite well with the ISPED thresholds based upon 75^th^ and 95^th^ percentiles of BMI at least between the ages of 5–17 years, consequently the international system proposed for inter-countries comparison and the Italian system have similar effects of on OW and OB classification and association with CMRFs. However, considering the seriousness of the obesity epidemic now under way, the results of our study arise an important question about whether the WHO standards, that allow to get the highest sensibility in identifying obese children/adolescents with clustered cardiometabolic risk factors, should be suggested instead of the more specific national standards, for clinical practice and obesity screening in Italy.

## Abbreviations

BMI: Body mass index
BP: Blood pressure
CMRF: Cardiometabolic risk factor
HDL-C: High-density lipoprotein-cholesterol
IFG: Impaired fasting glucose
IOTF: International Obesity Task Force
ISPED: Italian Society for Pediatric Endocrinology and Diabetology
K: Kappa
OB: Obesity
OR: Odds ratio
OW: Overweight
Tg: Triglycerides
WHO: World Health Organization
